# Genetic Properties Responsible for the Transgressive Segregation of Days to Heading in Rice

**DOI:** 10.1534/g3.119.201011

**Published:** 2019-03-20

**Authors:** Yohei Koide, Shuntaro Sakaguchi, Takashi Uchiyama, Yuya Ota, Ayumi Tezuka, Atsushi J. Nagano, Seiya Ishiguro, Itsuro Takamure, Yuji Kishima

**Affiliations:** * Research Faculty of Agriculture, Hokkaido University, Kita-9 Nishi-9, Kita-ku, Sapporo, 060-8589, Japan; † Faculty of Agriculture, Ryukoku University, 1-5 Yokotani, Seta Oe-cho, Otsu, Shiga 520-2194, Japan

**Keywords:** rice, transgressive segregation, extreme phenotype, days to heading, QTL

## Abstract

Transgressive segregation produces hybrid progeny phenotypes that exceed the parental phenotypes. Unlike heterosis, extreme phenotypes caused by transgressive segregation are heritably stable. We examined transgressive phenotypes of flowering time in rice, and revealed transgressive segregation in F_2_ populations derived from a cross between parents with similar (proximal) days to heading (DTH). The DTH phenotypes of the A58 × Kitaake F_2_ progenies were frequently more extreme than those of either parent. These transgressive phenotypes were maintained in the F_3_ and F_4_ populations. Both A58 and Kitaake are *japonica* rice cultivars adapted to Hokkaido, Japan, which is a high-latitude region, and have a short DTH. Among the four known loci required for a short DTH, three loci had common alleles in A58 and Kitaake, implying there is a similar genetic basis for DTH between the two varieties. A genome-wide single nucleotide polymorphism (SNP) analysis based on the F_4_ population identified five new quantitative trait loci (QTL) associated with transgressive DTH phenotypes. Each of these QTL had different degrees of additive effects on DTH, and two QTL had an epistatic effect on each other. Thus, a genome-wide SNP analysis facilitated the detection of genetic loci associated with extreme DTH phenotypes, and revealed that the transgressive phenotypes were produced by exchanging the complementary alleles of a few minor QTL in the similar parental phenotypes.

The range of phenotypic variation in a quantitative trait depends on the genetic complexity (Alonso-Blanco and Mendez-Vigo 2014; Huang and Han 2014). Hybridizations often produce progenies with wider phenotypic variation than their parents, which is referred to as transgressive segregation (Rick and Smith 1953; Harlan 1976; De Vicente and Tanksley 1993). Unlike heterosis, the extreme phenotypes that occur as a result of transgressive segregation can be fixed after the second filial generation (F_2_). Such extreme phenotypes can have important roles in evolution (Rieseberg *et al.* 2002; Dittrich-Reed and Fitzpatrick 2013). From a breeding perspective, this phenomenon has also strongly contributed to crop and animal improvements (Vega and Frey 1980; Tanksley and Mccouch 1997). However, little is known about the genetic basis of transgressive segregations.

Days to heading (DTH) determines the regional adaptability of rice (*Oryza sativa* L.), which is cultivated widely in tropical and temperate regions (Hori *et al.* 2016). Additionally, DTH is an important agronomic trait that controls flowering time in rice. Flowering time is a complicated trait in many crops, and the genetic basis of DTH has been well studied in rice; to date, 14 quantitative trait loci (QTL) have been identified based on natural variation and isolated with map-based cloning strategies (Ebana *et al.* 2011; Hori *et al.* 2016; Brambilla *et al.* 2017). We previously examined DTH in rice using six F_2_ populations derived from crosses between Kokusyokuto-2 (a Hokkaido landrace denoted as A58) with a short DTH (81 days) as the seed parent and six varieties with a long DTH (114–126 days) as the pollen parents (Ota *et al.* 2014). Most F_2_ plants from all six crosses had an intermediate DTH that fell within the parental ranges. Ota *et al.* (2014) detected some plants in the A58 × Kasalath F_2_ population with a shorter DTH than that of A58. Additionally, only this F_2_ population had some individuals with a shorter DTH relative to those of the parents, and the other five F_2_ populations did not exhibit such extreme phenotypes. Among the plants with a shorter DTH, we identified a genetic interaction (*Ghd7* from A58 and *Ehd1* from Kasalath) that contributed to the extreme phenotypes produced by the cross of the distantly related parents (Ota *et al.* 2014).

In the current study, we were interested in how the range of phenotypic variation is produced and whether extreme phenotypes can be produced when both parents in a cross have similar (proximal) phenotypes. We expected the progenies from parents with the identical genotypes to exhibit a very narrow phenotypic variation, while some phenotypic variation (*e.g.*, transgressive phenotype) was predicted for the F_2_ generation resulting from parents with diverse genotypes that can coincidentally cause similar phenotypes (De Vicente and Tanksley 1993; Mansur *et al.* 1993; Kim and Rieseberg 1999). By testing these predictions, we may be able to address the unknown genetic entities that produce extreme phenotypes.

We specifically focused on phenotypic variation in the DTH of a population derived from a cross between two closely related varieties, A58 and Kitaake (an improved variety), both of which are adapted to Hokkaido, the northernmost rice cultivation area in Japan. Compared with the progenies of the parents with different (distal) DTH phenotypes, extremely short or long DTH relative to those of the parents were more common among the A58 × Kitaake progenies. We evaluated the genetic causes of the transgressive segregations of both early and late DTH observed in this segregating population. First, known genes associated with a short DTH were evaluated in A58, Kitaake, and their progenies to determine if transgressive phenotypes were produced. Subsequently, we completed a genome-wide analysis of single nucleotide polymorphisms (SNPs) to detect previously undetected QTL associated with extremely short or long DTH. The results described herein demonstrated that a relatively small number of minor QTL and their epistatic interactions produced transgressive segregation in DTH. Moreover, important genetic properties of the extreme heading phenotypes caused by transgressive segregation are discussed.

## Materials and methods

### Plant materials and experimental design

A rice landrace from Hokkaido, A58, and an improved variety from Hokkaido, Kitaake, were used as parents. The A58 seeds were obtained from seed stocks at the Plant Breeding Laboratory of Hokkaido University. Kitaake seeds were obtained from the genebank at the Agricultural Research Department of the Hokkaido Research Organization. Additionally, A58 was crossed with Kitaake to obtain F_1_ seeds. A total of 248 F_2_ plants were obtained from the self-pollination of the F_1_ plants. Of the 248 F_2_ plants, 132 were randomly selected to generate 132 F_3_ lines. The genetics underlying the DTH of the F_3_ lines were analyzed with DNA markers in the *Hd1* locus, which is a major locus affecting the DTH in rice (Yano *et al.* 2000). Of the 132 F_3_ lines, 15 with the shortest DTH and either the A58- or Kitaake-type homozygous allele at the *Hd1* locus were selected as early-heading lines. Similarly, 15 lines with the longest DTH and either of the *Hd1* homozygous alleles were selected as late-heading lines. Plants from the early- and late-heading lines were self-pollinated to produce F_4_ lines, after which the genotypes of the resulting F_4_ lines were determined by a genome-wide SNP analysis (Supplementary Figure S1, File S1_genotype data).

### Days to heading analysis

Seeds were sown in early May, and 1-month-old plants were transplanted and grown in an experimental paddy field at Hokkaido University, Sapporo, Japan (43.1 N). For the F_2_ and F_3_ populations, DTH was measured in 2013 and 2014, respectively (File S2_phenotype data). The DTH for the F_4_ generation was measured in 2015 and 2016 as the number of days from sowing to the emergence of the first panicle (File S2_phenotype data). For 30 lines, the average DTH of each line was calculated from the values of five or six plants per line (File S2_phenotype data).

### Transgressive index

We calculated the transgressive index, which indicates the proportion of phenotypic differences between both parents and the phenotypic range in the F_2_ population. This index was calculated by dividing the difference between the maximum and minimum DTH in the F_2_ population by the parental DTH difference.

### Genotyping and sequencing

Three plants from each of 30 lines in the F_4_ population were independently subjected to the following procedures. Genomic DNA was extracted from leaf samples with Plant DNAzol (Invitrogen, Carlsbad, CA, USA). To genotype the *Hd1* locus, the following two primers were designed based on the *Hd1* sequence: Hd1L (5′-CGA CGT GCA GGT GTA CTC CG-3′) and Hd1R (5′-AAT CTG TGT AAG CAC TG ACG-3′). Genome-wide SNPs were detected via a double digest restriction site-associated DNA sequencing (ddRAD-Seq) (Baird *et al.* 2008; Peterson *et al.* 2012) analysis involving a DNA library that was prepared following a digestion with the restriction enzymes *Bgl*II and *Eco*RI. The library was sequenced with a HiSeq 2000 Sequencer (Illumina, San Diego, CA, USA) by Macrogen (Seoul, South Korea), with 51-bp single-end reads in one lane. The ddRAD-Seq reads were trimmed with Trimmomatic (version 0.33) (Bolger *et al.* 2014) with the following parameters: LEADING:19 TRAILING:19 SLIDINGWINDOW:30:20 AVGQUAL:20 MINLEN:51. Moreover, the default parameters of the Bowtie 2 program were used to map the trimmed reads to a RAD reference for the Os-Nipponbare-Reference-IRGSP-1.0 genome assembly (Langmead and Salzberg 2012). To generate the RAD loci, we used the ref_map.pl pipeline in Stacks (version 1.29) (Catchen *et al.* 2011). The ddRAD-Seq analysis was completed by Clockmics, Inc. (Izumi, Osaka, Japan). A total of 1,402 SNPs between parental varieties were detected by ddRAD-Seq; among these SNPs, 634 were considered reliable after filtering SNPs that appeared in more than 80% of F_4_ plants (File S1_genotype data).

The amplicons for the four previously identified DTH-related genes (*Hd1*, *Hd2*/*OsPRR37*, *E1*/*Hd4*/*Ghd7*, and *Hd5*/*DTH8*) were purified with the NucleoSpin Gel and PCR Clean-up kit (Macherey-Nagel, Düren, Germany). The purified samples were sequenced in both directions with the BigDye Terminator Cycle Sequencing kit (Applied Biosystems, Foster City, CA, USA) and the ABI 310 automatic sequencer (Applied Biosystems). Sequences were aligned with CLUSTAL W 2.1 (Thompson *et al.* 1994). The following primers were designed to sequence the four genes: *Se1*/*Hd1* [5′-CGA CGT GCA GGT GTA CTC CG-3′ and 5′-AAT CTG TGT AAG CAC TG ACG-3′], *Hd2*/*OsPRR37* [5′-TCT TTC TGA TGG CTG TCT GC-3′ and 5′-GCC ATC GCG TAG GTA GGT AG-3′], *E1*/*Hd4*/*Ghd* [5′-GCT GGC TGG ACT TCA CTA CC-3′ and 5′-CAT GGG CCA CTT CTA AGA TCA-3′], and *Hd5*/*DTH8* [5′-CGG AGT TCA TCA GCT TCG TT-3′ and 5′-TGA CCA TGG TGT GAG TGT GA-3′].

### Marker genotype value

The allelic effects of each of the six loci influencing the DTH of the A58 × Kitaake hybrid progenies were evaluated as marker genotype values (MGVs) (Goddard and Hayes 2007). The average DTH for each allele was calculated based on the DTH data collected for all homozygous alleles in the 30 F_4_ lines in 2016. The average was determined based on the two phenotypic averages obtained for each homozygous allele at the same locus. The MGV that expressed the effect of SNP locus was the difference between the average DTH of all samples and the DTH of either allele, which was equivalent to a half of the difference between the DTHs of both alleles at the same locus, and was calculated with the following equation: A = (B − C)/2; where A: MGV (absolute value), B: average DTH of individuals carrying either homozygous allele, and C: average DTH of individuals carrying the other homozygous allele. Increasing MGVs corresponded with increasing allelic effects at the locus.

### Data availability

All genetic stocks and sequence data are available on request. A set of 90 ddRAD-Seq data from the 30 lines and the whole genome sequence data of A58 generated in this study were deposited in the DDBJ database (accession number DRA008112 and DRA007777, respectively). The other whole genome data for Kitaake, and Nipponbare are available in accession number SRA054074 at DDBJ (https://www.ddbj.nig.ac.jp/index-e.html), and IRGSP-1.0 at RAP-DB (https://rapdb.dna.affrc.go.jp/download/irgsp1.html), respectively. A total of 634 SNP genotypes for 15 early- and 15 late-heading lines at F_4_ are listed in File S1_genotype data, and DTH data of 15 early- and 15 late-heading lines at F_3_ (2014) and F_4_ (2015 and 2016) are listed in File S2_phenotype data. Supplemental material available at https://doi.org/10.25387/g3.7506041.

## Results

### Variations and transgressive DTH phenotypes in A58 × Kitaake progenies

**Figure 1:**
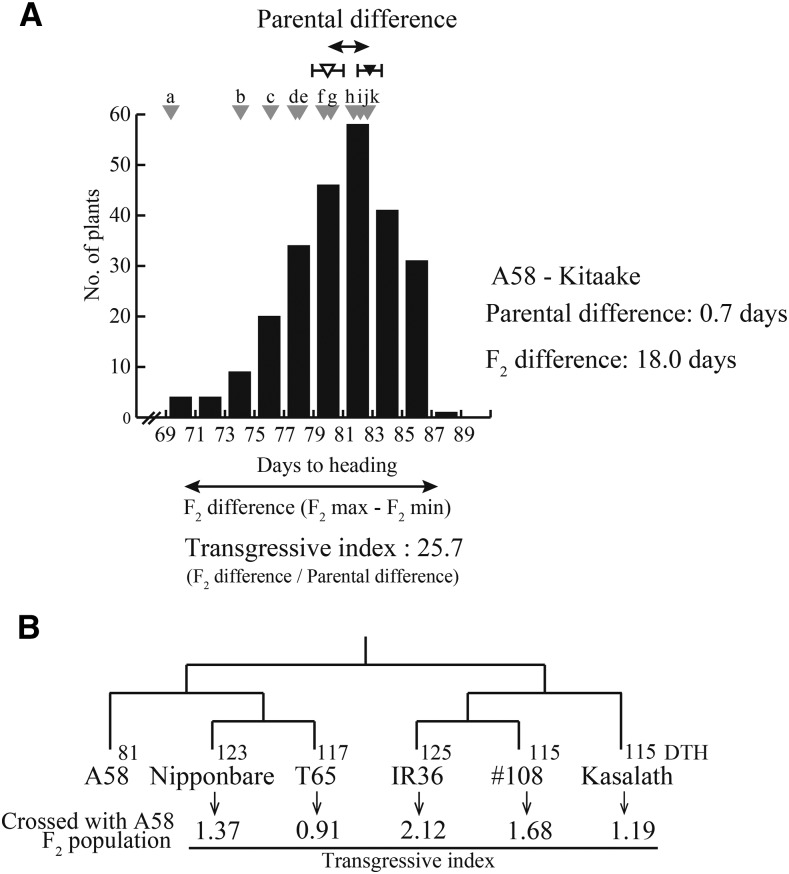
Transgressive segregation and genetic relationships between parental varieties. (A) Frequency distribution of DTH for A58 × Kitaake F_2_ plants. The transgressive index represents the ratio of the F_2_ population DTH distribution to the parental difference. The DTH difference between A58 and Kitaake was 0.7 days, and the DTH range in the F_2_ population was 18.0 days, which resulted in a transgressive index of 25.7. The DTH for A58 and Kitaake are represented by filled and empty arrowheads, respectively. Standard DTH values for the following 11 varieties grown in Hokkaido are indicated by gray arrowheads: (a) Kitaibuki, (b) Hakucho-mochi, (c) Daichinohoshi (d) Hatsushizuku, (e) Hoshinoyume, (f) Kuiku180, (g) Hokuiku-mochi, (h) Nanatsuboshi, (i) Kirara397, (j) Hoshimaru, and (k) Gimpu. (B) Transgressive indices for crosses between A58 and each of five other varieties. The phylogenetic relationships and the associated dendrogram for the five *O. sativa* varieties, Nipponbare (*japonica*), T65 (*japonica*), IR36 (*indica*), #108 (*indica*), and Kasalath (*indica*, Aus), are presented based on information provided in Takata *et al.* (2005). The DTH for each variety is also indicated. To calculate the transgressive index, the DTH of the parental varieties and F_2_ plants were calculated based on data from Ota *et al.* (2014). For all five combinations, 93 F_2_ plants were examined (Ota *et al.* 2014).

Both A58 and Kitaake are adapted to the high-latitude area between 41.2 N and 45.4 N in Hokkaido, Japan, and consequently are cold-resistant and have a photoperiod-insensitive, short DTH (Ishiguro *et al.* 2014; Ota *et al.* 2014). There was no significant difference in the DTH of these two varieties (*t*-test: A58, 81.2 ± 0.38; Kitaake, 80.5 ± 0.66; *P* = 0.19) (Figure 1A). The DTH of the F_2_ plants of the two varieties ranged from 69 to 87 days (Figure 1A), and the earliest DTH was equivalent to that of an extreme early-heading variety (Figure 1A).

The transgressive index of the A58 × Kitaake progenies was 25.7 (Figure 1A), indicating that DTHs of many plants of the A58 × Kitaake F_2_ population exceeded those of a range between their parents. Such a strong transgressive segregation was not observed for previously published crosses (Ota *et al.* 2014). In the six crosses between A58 and the other varieties (Takata *et al.* 2005; Ota *et al.* 2014), the transgressive indices ranged from 0.91 to 2.12 (Figure 1B). The genetic distances among the three *japonica* varieties (A58, Kitaake, and Nipponbare) were estimated based on the number of SNPs, which was obtained by comparing the whole genome sequences; accessions DRA007777 (DDBJ), SRA054074 (DDBJ), and IRGSP-1.0 (RAP-DB). We detected 288,500 SNPs between A58 and Nipponbare, 294,982 SNPs between A58 and Kitaake, and 202,776 SNPs between Kitaake and Nipponbare. From smallest to largest, the genetic distances were as follows: Kitaake – Nipponbare < A58 – Nipponbare < A58 – Kitaake. Therefore, transgressive segregation was not directly affected by the genetic distance between parents, but by phenotypic similarity instead.

The DTH distribution for the A58 × Kitaake-derived F_3_ population formed a unimodal curve similar to that of the F_2_ population, and shifted to 10 days earlier than the F_2_ population because of differences in the weather conditions or some circumstantial conditions (Supplementary Figure S2). For a further analysis, we selected 15 early- and late-heading F_3_ plants and developed two F_4_ populations (early and late) by self-pollination. The average DTH of the early- and late-heading F_4_ populations were 63.8 ± 1.32 and 74.6 ± 0.99, respectively, in 2015, and 72.2 ± 1.32 and 80.0 ± 1.00, respectively, in 2016 (Supplementary Figure S3, File S2_phenotype data). These differences in the DTH of the early- and late-heading populations were significant (*t*-test, *P* < 0.001) in 2015 and 2016, suggesting that these two distinct populations were generated by new genetic combinations or interactions derived from the A58 × Kitaake cross.

### Sequence analysis of genes controlling DTH, and the effect of Hd1 on DTH

**Figure 2:**
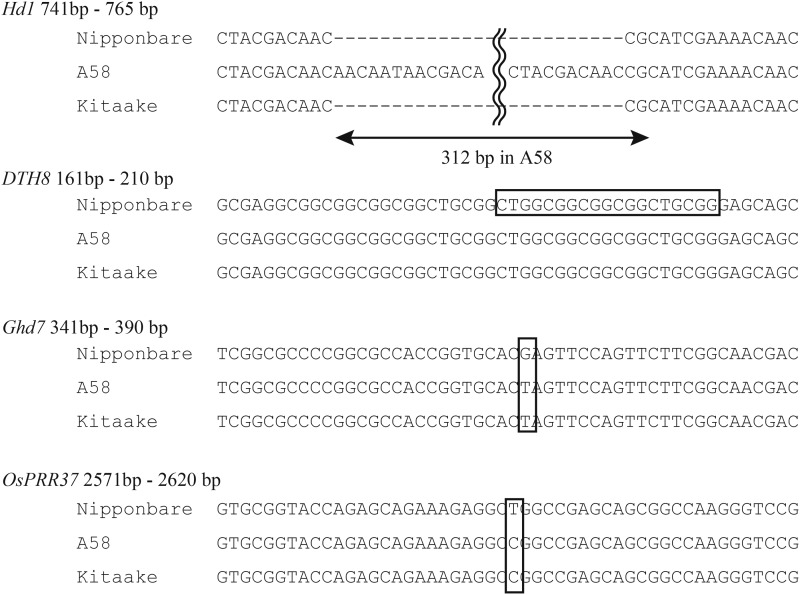
Comparisons of Nipponbare, A58, and Kitaake regarding the partial nucleotide sequences of four major loci affecting DTH in Hokkaido. The sequenced positions (based on Nipponbare) were selected using known polymorphisms among varieties grown in Hokkaido that were observed in previous studies (Ichitani *et al.* 1997; Fujino and Sekiguchi 2005a; Fujino and Sekiguchi 2005b; Nonoue *et al.* 2008; Fujino *et al.* 2013; Koo *et al.* 2013). Multiple differences in *Hd1* were detected between A58 and Kitaake. Kitaake possesses a functional allele that is also present in Nipponbare. In *DTH8*, a 19-bp segment (indicated by a rectangle) was deleted in most of the other Hokkaido varieties, but not in Nipponbare, A58, and Kitaake, for which we could not detect any polymorphisms. For *Ghd7* and *OsPRR3*7, the SNPs observed in Nipponbare and the other two varieties are indicated by boxes.

Four loci (*E1*/*Hd4*/*Ghd7*, *Hd2*/*OsPRR37*, *Se1*/*Hd1*, and *Hd5*/*DTH8*) control DTH in rice varieties from Hokkaido, and their specific alleles produced photoperiod-insensitive varieties with a short DTH that were adapted to the environmental conditions in northern Japan (Ichitani *et al.* 1997; Fujino and Sekiguchi 2005a; Fujino and Sekiguchi 2005b; Nonoue *et al.* 2008; Fujino *et al.* 2013; Koo *et al.* 2013; Gao *et al.* 2014). To confirm whether these four loci are related to the DTH differences observed in the A58 × Kitaake F_2_ population, we compared the nucleotide sequences of these loci (Figure 2). An analysis of the *Hd1* sequence revealed the presence of polymorphisms, including a 312-bp insertion/deletion in A58 and Kitaake. This polymorphism in *Hd1* might have been responsible for the DTH differences observed in the F_2_ population. In contrast to *Hd1*, the other three loci (*E1*/*Hd4*/*Ghd7*, *Hd2*/*OsPRR37*, and *Hd5*/*DTH8*) lacked polymorphisms in A58 and Kitaake (Figure 2).

Regarding the effect of the *Hd1* locus on the A58 × Kitaake F_2_ population, the average DTH in the A58-type homozygous, heterozygous, and Kitaake-homozygous populations were 81.3 ± 0.36, 79.5 ± 0.38, and 78.8 ± 0.46, respectively (Table 1). The results implied that *Hd1* had a significant, but small, effect on the DTH in this population (*P* < 0.001) (Table 1), indicating the extremely early-heading phenotype of the progenies was not solely due to *Hd1*.

**Table 1:** The effect of Hd1 locus on days to heading in F2 population derived from A58 x Kitaake cross

Marker			No. of plants				Average DTH			
name	Chr.	Position	A58-type	Heterozygous	Kitaake-type	P*	A58-type	Heterozygous	Kitaake-type	P**
Hd1	6		56	103	73	6 x 10^−2^	81.3	79.5	78.8	3 x 10^−5^

* *P* value for the probability of Mendelian segregation of genotypes obtained by a Chi squared test (1:2:1 ratio).

** *P* value for the probability determined by T-test.

### Detection of SNPs associated with extreme DTH phenotypes

If a QTL for DTH was located near a SNP, the SNP alleles tended to be associated with early- or late-heading populations. A genome-wide SNP analysis by ddRAD-Seq resulted in the identification of 634 reliable SNPs for the 15 early-heading and 15 late-heading lines in the F_4_ population (Supplementary Figure S4, File S1_genotype data). Among the 634 SNPs, 27 were detected at loci where the frequency of either the A58- or Kitaake-type homozygous allele was distorted from a 1:1 segregation in the early- and late-heading populations (*P* < 0.05) (Supplementary Table S1). Of these possible DTH-related SNPs, we focused on 19 SNPs, which were grouped into five clusters on chromosomes 1, 2, 4, 6, and 10 (Supplementary Table S2 and Supplementary Figure S4). Each cluster had multiple SNPs within 3.5 Mb, which might not only contain single QTL for DTH, but more QTL. For each SNP locus in these clusters, the average DTH for A58- and Kitaake-type homozygous alleles and MGVs were calculated for the F_4_ population in 2015 and 2016 (Supplementary Table S2).

### Validation of relationships Between SNP genotypes and DTH

**Figure 3:**
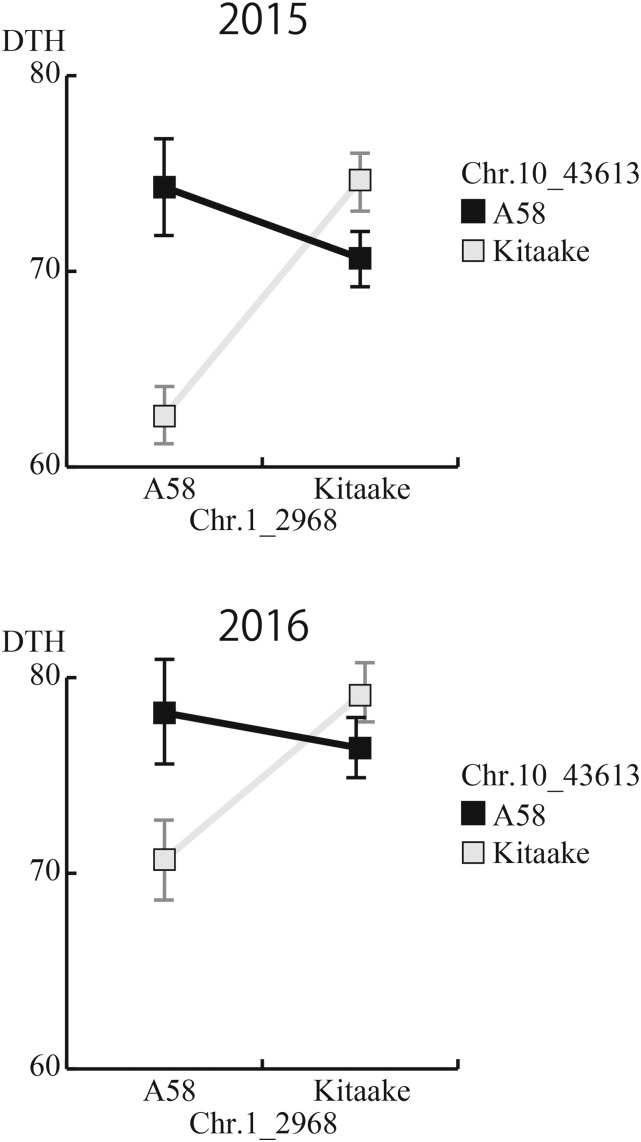
Epistatic interaction between SNPs on chromosomes 1 and 10 influencing the DTH observed in 2015 and 2016. Average DTH values for the four combinations of genotypes with SNPs (C1_2301 and C10_43613) with the highest MGV in the clusters on chromosomes 1 and 10, which are indicated by squares. Chromosomes 1 and 10 were selected from all combinations involving chromosomes 1, 4, 6, and 10 (Supplementary Figure S6). When the A58 SNP on chromosome 10 (black line) and the Kitaake SNP on chromosome 10 (gray line) were respectively coupled with different parental SNPs, epistatic (allelic) interactions occurred. In particular, the combination of the A58 allele on chromosome 1 and the Kitaake allele on chromosome 10 resulted in the shortest DTH.

The average DTH for the A58- and Kitaake-type homozygous alleles of each of the five SNP clusters in the F_4_ lines examined in 2015 and 2016 are presented in Supplementary Table S2. The highest MGVs (5.9–4.1) were calculated for SNPs on chromosome 4 in 2015 and 2016 (Supplementary Table S2 and Supplementary Figure S5). For the SNP cluster on chromosome 10, the MGVs were 3.8–2.4. Meanwhile, the MGVs for the SNPs on chromosomes 1 and 6 were 2.9–1.7 and 3.1–0.7, respectively. Among the five clusters, the weakest effect was detected for the chromosome 2 cluster, with MGVs of 1.9–1.7 (Supplementary Table S2). Although the overall values were lower in 2016 than in 2015, the order of the DTH MGVs for the five SNP clusters was consistent (*i.e.*, chromosome 4 > 10 > 6 > 1 > 2 > *Hd1*). The Kitaake-derived alleles for the SNPs on chromosomes 4, 10, and 6 produced a shorter DTH than the A58-derived alleles. In contrast, the A58-derived alleles for the SNPs on chromosomes 1 and 2 produced a shorter DTH than the Kitaake-derived alleles (Figure 3).

The F_2_ population was analyzed for genetic interactions among the selected chromosomal regions (chromosomes 1, 4, 6, and 10) (Figure 1). Of the several combinations of possible epistatic interactions, a strong genetic interaction was identified for the SNPs on chromosomes 1 and 10 (Figures 3 and Supplementary Figure S6). The A58-derived alleles associated with the SNPs on chromosome 1 decreased the DTH when they were combined with Kitaake-derived alleles associated with SNPs on chromosome 10, but had the opposite effect when combined with A58-derived alleles associated with SNPs on chromosome 10 (Figure 3). No known gene associated with DTH was detected around these two chromosomal regions. These findings revealed that unknown genes are responsible for epistatic interactions underlying the transgressive early-heading phenotype.

**Figure 4:**
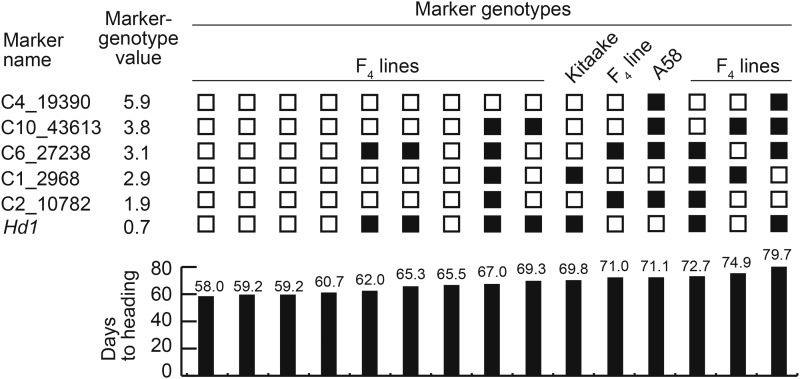
Phenotypic relationships with six marker genotype value combinations. Among the 30 F_4_ lines, 13 retained the homozygous alleles in the six loci corresponding to the SNP clusters with QTL for DTH and *Hd1*. The effect of each locus on DTH was weighted according to marker genotype values (see Materials and Methods) based on the DTH in 2015. Larger values indicate a stronger effect on DTH. Empty squares indicate smaller DTH effects relative to those of black squares. Kitaake contained four alleles for shorter DTH in chromosome 4, chromosome 10, chromosome 6, and chromosome 2, whereas A58 possessed two alleles for shorter DTH in chromosome 1 and *Hd1*. The two parental cultivars, Kitaake and A58, had DTH of 69.8 and 71.1 days, respectively. The DTH in the selected F_4_ lines ranged from 58.0 to 79.7 days. Each marker name represents the central SNP in the cluster.

Loci weighted by MGVs based on DTH data from 2015 are shown in Figure 4. Of 30 F_4_ lines, 13 harbored homozygous alleles at all six loci (the five QTL and *Hd1*). These 13 lines were sorted by DTH, and analyzed based on specific factors, including MGVs, direction of the allelic effect, and the number of alleles with an effect (Figure 4). The lines with a short DTH tended to have more alleles with a short DTH effect, whereas the lines with a long DTH contained more alleles with the opposite effect. Therefore, the extreme phenotypes produced by transgressive segregation might be defined by the allelic composition, with different phenotypic effects occurring in either direction. However, the order of the 13 lines based on DTH was somewhat inconsistent with the overall evaluations of these factors. This discrepancy may have been due to unknown genes and/or undetected genetic interactions.

## Discussion

In this study, we determined that transgressive segregation occurred in the hybrid progenies of two rice varieties, A58 and Kitaake, both of which were able to adapt to a high-latitude region in part because of a short DTH. Phenotypic variation beyond the parental range were observed in this segregating population and helped to uncover the genetic basis of transgressive segregation and extreme DTH phenotypes. The two parental varieties shared the same genotypes for three known major QTL (*E1*/*Hd4*/*Ghd7*, *Hd2*/*OsPRR37*, and *Hd5*/*DTH8*) (Figure 2), but different alleles for *Se1*/*Hd1* and several unknown minor QTL. The different genotypes in minor QTL produced new genetic combinations that resulted in the transgressive phenotypes of the progenies. Quantitative trait loci direct either positive or negative actions based on the effect of parental alleles. If negative QTL alleles in either parent are replaced by the positive alleles of the other parent, the progeny may obtain the desired phenotype because of the presence of more positive alleles (De Vicente and Tanksley 1993; Xu *et al.* 1998; Rieseberg *et al.* 1999). Our results appeared to confirm this scenario, as we observed allelic complementation at QTL, and our data revealed the importance of such “hidden” genetic variations despite close phenotypic relationships (Hagiwara *et al.* 2006; Mao *et al.* 2011).

We analyzed SNPs by deep sequencing to obtain a sufficient number of markers for the closely related varieties. This approach resulted in detection of more than 600 genome-wide SNPs between both Hokkaido-adapted varieties (Supplementary Figure S4). Additionally, the similar genetic backgrounds of the two varieties, A58 and Kitaake, facilitated the identification of the minor QTL that shape transgressive early heading by genome-wide SNP analysis.

We detected five SNP clusters corresponding to QTL and the *Hd1* locus, which contributed to DTH differences in the A58 × Kitaake progenies (Tables 1 and Supplementary Table S2). These QTL were involved in both the additive and epistatic effects on extreme heading phenotypes (Supplementary Table S2 and Figure 3). Among the SNP clusters, the strongest effect was explained by the chromosome 4 cluster, in which the Kitaake-derived allele(s) decreased the DTH (Supplementary Table S2). This cluster was located from 29.8 to 32.4 Mb on chromosome 4 (Supplementary Figure S4), where only one gene, *Rice FLO-LFY homolog* (*RFL*) (Kyozuka *et al.* 1998), is functionally characterized as a flowering-related gene in the QTL Annotation Rice Online (Q-TARO) database (http://qtaro.abr.affrc.go.jp/). Similarly, the chromosome 6 cluster (from 24.5 to 25.5 Mb) included a gene for photoperiod sensitivity, *Se5* (Izawa *et al.* 2000). However, neither synonymous or nonsynonymous polymorphism in A58 and Kitaake were detected in either *RFL* or *Se5*. The other QTL detected in the SNP clusters on chromosomes 1, 2, and 10 lacked any known DTH-related genes. These results imply that some unknown genes present in these SNP clusters affect the DTH of the Hokkaido varieties. Interestingly, our analysis also revealed possible epistatic interactions between genes in the SNP clusters on chromosomes 1 and 10 that shortened the DTH (Figure 3). It was previously thought that epistasis is unlikely to be a major cause of transgressive phenotypes (De Vicente and Tanksley 1993; Rieseberg *et al.* 1999); however, in our study, epistatic interactions explained the transgressive phenotypes observed in the segregating populations (Figure 3).

To date, four genes (*E1*/*Hd4*/*Ghd7*, *Hd2*/*OsPRR37*, *Se1*/*Hd1*, and *Hd5*/*DTH8*) were reported to control DTH in the improved rice varieties cultivated in Hokkaido (Ichitani *et al.* 1997; Fujino and Sekiguchi 2005a; Fujino and Sekiguchi 2005b; Nonoue *et al.* 2008; Fujino *et al.* 2013; Koo *et al.* 2013; Gao *et al.* 2014). Among the four genes, loss-of-function alleles in *E1*/*Hd4*/*Ghd7* and *Hd2*/*OsPRR37* are necessary for photoperiod insensitivity in rice varieties grown in northern areas (Fujino and Sekiguchi 2005a; Fujino and Sekiguchi 2005b; Xue *et al.* 2008; Koo *et al.* 2013; Gao *et al.* 2014). Alternatively, the other two genes (*Se1*/*Hd1* and *Hd5*/*DTH8*) have small effects on photoperiod insensitivity among the varieties grown in Hokkaido (Ichitani *et al.* 1997; Fujino and Sekiguchi 2005b; Nonoue *et al.* 2008). In this study, analyses of *Se1*/*Hd1* sequences revealed differences between A58 and Kitaake. Specifically, A58 has insertion/deletion mutations, whereas Kitaake carries the functional allele (Figure 2). Both varieties had the same loss-of-function alleles at the *E1*/*Hd4*/*Ghd7* and *Hd2*/*OsPRR37* loci and had the same functional allele at the *Hd5*/*DTH8* locus (Figure 2). These results indicate that the improved varieties in Hokkaido might have inherited the same *E1*/*Hd4*/*Ghd7*, *Hd2*/*OsPRR37*, and *Hd5*/*DTH8* alleles from a landrace similar to A58, which facilitated adaptations, because photoperiod insensitivity was essential for adaptation. In addition to these four loci, newly identified minor QTL for DTH were identified on chromosomes 1, 2, 4, 6, and 10 (Supplementary Table S2 and Supplementary Figure S4). These QTL likely contributed to the extreme DTH phenotypes produced by transgressive segregation based on the composition of their complementary alleles (Figure 4).

**Figure 5:**
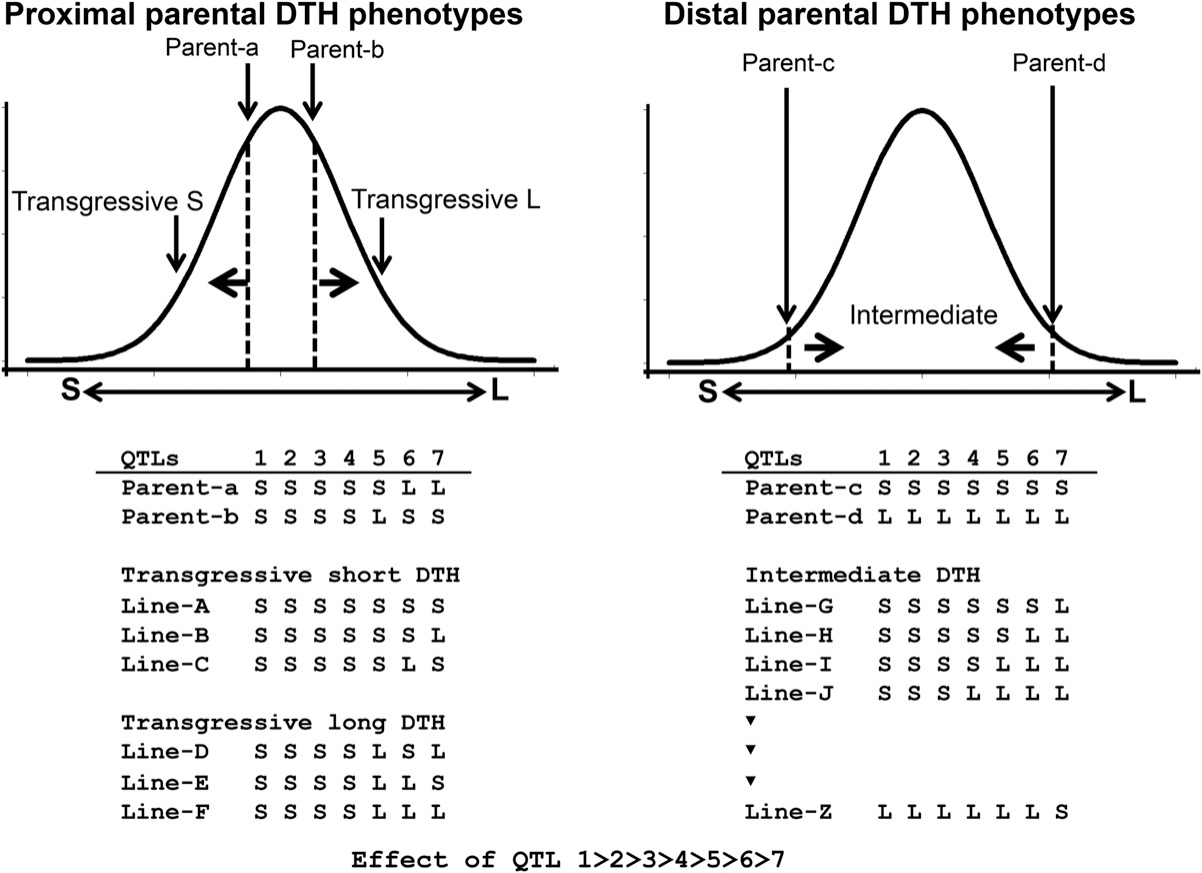
Model of different segregation patterns that occurred in the F2 populations derived from two parental combinations of proximal and distal DTH phenotypes. The left panel represents the segregation pattern of the F_2_ population derived from a cross between parent-a and parent-b with proximal DTH phenotypes due to the similar genotypes. Because of differences in a few alleles with minor effects on DTH, the F_2_ progenies produced transgressive phenotypes. The right panel represents the F_2_ population produced by parents with distal phenotypes and the opposite genotypes, revealing the intermediate segregation between both parents. Most of the F_2_ progenies with mixed genotypes of the parental alleles did not have DTH phenotypes that exceeded those of the parents. There are seven loci influencing the DTH, and their effects on DTH are ordered as 1 >>> 7. Additionally, S and L indicate the effect of an allele at each locus that makes the DTH shorter or longer, respectively.

Rieseberg *et al.* (1999) made several predictions regarding the cause of transgressive segregation, one of which is consistent with our results. Specifically, transgressive segregation is likely in the F_2_ population derived from parents with more proximal phenotypes (Figure 5). Among the known alleles at the four loci that are necessary for DTH adaptations to Hokkaido, the same alleles were detected at three loci (*i.e.*, not *Hd1*) in A58 and Kitaake. These findings indicate that these two varieties possess many common alleles that shorten DTH. Our results demonstrate that transgressive segregation mainly occurred as a result of a few unknown QTL, in which alleles combined in a complementary manner (Figure 5).

According to Rieseberg *et al.* (1999), transgressive segregation tends to occur more frequently in intraspecific crosses, inbred populations, and domesticated populations than in interspecific crosses, outbred populations, and wild populations, respectively. The lack of a strong positive correlation was observed between parental genetic divergence and transgression frequency (Rieseberg *et al.* 1999; Rieseberg *et al.* 2003). However, in terms of interspecific F_1_ hybrids, Stelkens and Seehausen (2009) analyzed the available published data regarding transgressive phenotypes and described a tendency for transgression frequency to be positively correlated with genetic distance, likely mainly because of an increase in complementary gene activity and epistasis. In our previous study involving F_2_ populations (Ota *et al.* 2014), we detected a lack of correlation between parental genetic divergence and transgression frequency for several cross combinations with varying genetic distances (Figure 1B). The hybrid progenies of two varieties with distal DTH adapted to different environments exhibited few instances of transgressive DTH phenotypes (Figure 1B). Because the parents with different genetic backgrounds (*e.g.*, interspecies) adapted to diverse environments, many new allelic combinations were generated in the hybrid progenies (Figure 5). Such complex allelic combinations might generate positive and negative genetic interactions, and offset allelic effects. Stochastically, if there are many segregating loci, individuals rarely accumulate only the alleles with positive effects, with most usually containing at least a few alleles with negative effects (Figure 5).

This study confirmed that a few genes and their combinations expanded the variability in the DTH phenotype of the progenies whose parents exhibit similar phenotypes. If many minor QTL influence a trait, a cross between parents with proximal phenotypes might result in more transgressive individuals than a cross between parents with distal phenotypes. Consequently, it might be useful to identify QTL or allelic interactions associated with the transgressive DTH phenotypes in progenies of other varieties with proximal phenotypes. Similarly, to integrate other transgressive phenotypes into breeding programs, a few alleles with the additive effects of minor and hidden QTL should be targeted in varieties with proximal phenotypes.
